# Patterns of Cellular Distribution with the Sentinel Node Positive for Breast Cancer

**DOI:** 10.4061/2011/873987

**Published:** 2011-09-08

**Authors:** Ekaterini Tsiapali, Marcia M. Schmidt, Don Dizon, Margaret Steinhoff, Jennifer Gass

**Affiliations:** Program in Women's Oncology, Women and Infants Hospital, 101 Dudley Street, Providence, RI 02905, USA

## Abstract

*Background*. Sentinel node biopsy (SNB) represents the standard of care in breast cancer axillary evaluation. Our study aims to characterize the patterns of malignant cell distribution within the sentinel nodes (SN). *Methods*. In a retrospective IRB-approved study, we examined the anatomic location of the nodal area with the highest radioactive signal or most intense blue staining (hot spot) and its distance from the metastatic foci. *Results*. 58 patients underwent SNB between January 2006 and February 2007. 12 patients with 19 positive SN were suitable for analysis. 4 (21%) metastases were located in the nodal hilum and 15 (79%) in the cortex. 6 (31%) metastases were found adjacent to the hotspot, and 9 (47%) within 4 mm of the hotspot. *Conclusions*. In our pilot series, SN metastases were within 4 mm of the hotspot in 78% of the cases. Pathologic analysis focused in that area may contribute to the more accurate identification of nodal metastases.

## 1. Introduction

The technique of sentinel node biopsy with lymphatic mapping has revolutionized breast cancer surgery, reducing the resultant risks of undesired consequences from more extensive axillary surgery and leading to the detection of increasingly smaller metastatic foci in the axilla. Surgeons strive to achieve a singular surgical intervention to the axilla by identifying those patients with positive sentinel nodes who would benefit from further nodal dissection. Several intraoperative techniques for lymph node evaluation are available including frozen section, touch imprint cytology, and RT-PCR breast lymph node (BLN) assay. These techniques have variable sensitivities and high specificity and involve different degrees of nodal tissue manipulation. 

We hypothesize that a better understanding of the distribution of the metastatic foci within the sentinel lymph node may aid in their more accurate detection and better utilization of intraoperative evaluation techniques.

## 2. Methods

The study was reviewed and approved by the IRB. Patients with invasive breast cancer who underwent sentinel node biopsy at our institution by a single surgeon between January 2006 and February 2007 were eligible for the study. The sentinel nodes were identified after the injection of intradermal radioactive Tc99-labeled sulfur colloid and subareolar methylene blue. 

The radioactive nodes were scanned with a gamma probe intraoperatively, and the area of highest radioactivity (hot spot) was identified and marked with a clip. Isotopically nonactive nodes were clipped at the site of maximum blue stain intensity. During subsequent routine pathologic examination, the hotspot was inked by the pathology technician, and the clip was removed. Data regarding the anatomic location of the hot spot and its distance from the metastatic foci in the positive sentinel nodes was collected. The charts were retrospectively reviewed.

## 3. Results

Fifty-eight patients with invasive breast cancer undergoing sentinel node biopsy for a clinically negative axilla at our institution by a single surgeon between January 2006 and February 2007 were identified, yielding a total of 127 removed sentinel nodes. Of these patients, 42 had a negative sentinel node biopsy and were excluded. In three cases, the clip marking the hot spot was not present at the time of pathologic examination. One other patient had a positive sentinel node that was completely replaced with metastatic disease. These four patients were also excluded from the study. Patients who had been identified by axillary ultrasound-guided fine-needle aspiration as node positive were not included in the study group.

A total of 12 patients with 19 positive sentinel nodes fulfilled inclusion criteria and were analyzed. The patient and tumor characteristics are listed in Table 1. 

**Figure 1:**
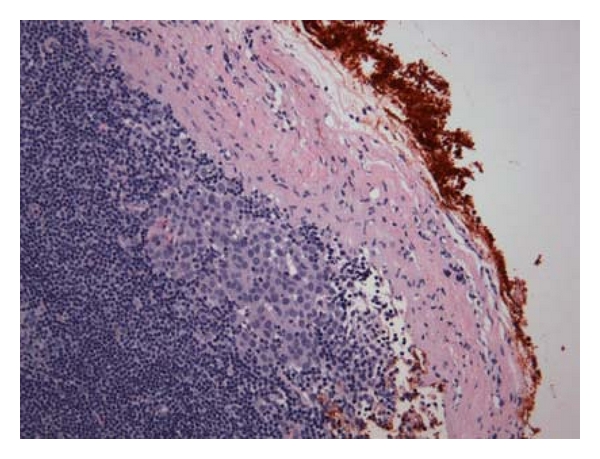
Micrometastases located in cortex close to hotspot.

**Figure 2:**
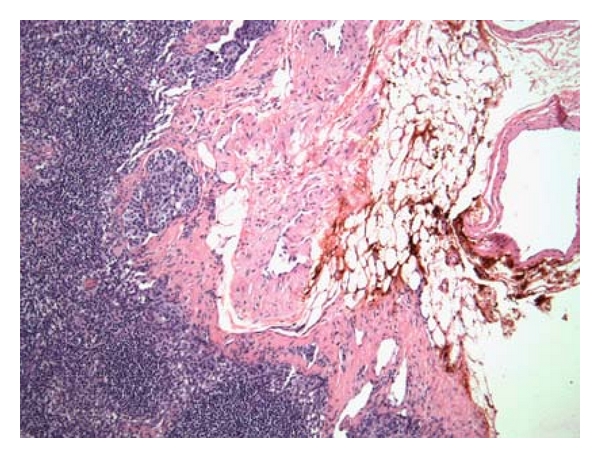
Micrometastases located in hilum close to hotspot.

The majority of the tumors were infiltrating ductal carcinomas (66.6%), and all were ER and/or PR positive. Of the 19 nodes evaluated, 3 (16%) were positive for isolated tumor cells (ITC), 8 (42%) contained micrometastases, and 8 (42%) macrometastases. The mean size of the examined positive lymph nodes was 10.9 mm, ranging from 5 mm to 21 mm and the mean size of the metastases 6.3 mm, ranging from ITC to 11 mm. The hotspot was located in the nodal cortex in 16 (85%) of the examined positive nodes. The 95 negative nodes in our cohort were also examined, and the hot spot was found in the cortex in 77 (81%) of nodes. In the nodes with tumor cells, the metastatic focus was located in the nodal cortex and adjacent medulla in 15 (79%) of the nodes (Figure 1) and in the nodal hilum in the remaining 4 (21%) nodes (Figure 2). Of the 4 nodes that had the metastatic focus in the hilum, one contained a micrometastasis and the other three macrometastases. The metastatic focus was located immediately adjacent to the marked hot spot in 6 (31%) nodes and within 4 mm of the hot spot in 9 (47%). Therefore, in 81% of the cases, the hotspot was located in the cortex, and in 78% of the cases the metastases were within 4 mm of the hotspot (Table 2).

## 4. Discussion

Sentinel node biopsy represents the standard of care for axillary evaluation in patients with breast cancer. The NSABP B-32 phase III clinical trial comparing sentinel lymph node biopsy to conventional axillary lymph node dissection in clinically node negative patients showed equivalent outcomes in overall survival, disease-free survival, and regional control with decreased morbidity in the sentinel node group [1, 2]. More recent data on patient-reported outcomes for sentinel lymph node biopsy versus axillary lymph node dissection showed that in the first six to twelve months axillary node dissection patients reported ipsilateral arm and breast morbidity, impaired quality of life, restricted work, and social activity more than the sentinel lymph node resection group. By twelve to thirty-six months, less than 15% in each group had any residual symptoms [3]. 

In patients found to have positive nodes on sentinel node biopsy, the standard of care has been to perform a completion axillary node dissection either at the time of the initial surgery or at a later date. In evaluating outcomes based on timing of the dissection, the ACoSOG Z-0010 and Z-0011 trials demonstrated that patients who undergo immediate as opposed to delayed completion axillary node dissection experience more short-term morbidity, but long-term outcomes were the same in both groups [4]. However, clearly, the duration to recovery is significantly greater when reoperation is required. 

In an effort to reduce reoperation, several techniques have been developed for intraoperative lymph node evaluation including frozen section, touch imprint cytology, and RT-PCR breast lymph node (BLN) assay. All techniques aim to accurately detect metastatic disease intraoperatively and allow the appropriate patient to proceed directly to axillary dissection. The sensitivity of touch imprint cytology hovers around 70% with a specificity of over 95% [5, 6]. High false negative rates have been noted in cases of micrometastases and invasive lobular carcinoma. Intraoperative frozen section, though reported to have similar sensitivity and specificity to touch imprint cytology, involves the undesirable loss of tissue for permanent section analysis, as well as longer turn around time and greater expense [7]. 

Several studies have been performed to evaluate genesearch real-time RT-PCR breast lymph node (BLN) assay in detecting intraoperative metastases in sentinel lymph nodes greater than 0.2 mm [8]. This method identifies mRNA from cytokeratin 19 (CK19) and mammaglobin(MG) genes expressed in epithelial cells but not present in lymphoid tissue. The nodal tissue is homogenized and using RT-PCR, and the mRNA is amplified and metastases identified. When compared to standard hematoxylin and eosin (H&E) staining, BLN achieves a sensitivity of 95.7% for macrometastases, 60.0% for micrometastases, and 55.6% for isolated tumor cells [9]. Though the accuracy of BLN assay is comparable to permanent section, it has not been rapidly adopted across the country with criticisms centered around the 30–40 minute turn around time, the specimen loss associated with tissue processing, the setup costs and the need for a skilled technician to perform the study. 

At this point, the indications for the use of all of these techniques are under reevaluation in view of recently published data from ACoSOG Z-0011 regarding the impact of completion axillary node dissection in patients with 1–3 positive sentinel nodes [10, 11]. This trial evaluated the impact of axillary dissection on both locoregional recurrence and overall survival in patients undergoing breast conservation therapy who were found to have one to three positive sentinel nodes, by randomizing patients to axillary dissection or no further surgery. Both groups were treated with adjuvant systemic therapy and whole breast radiotherapy, and, after six years of follow-up, there was no difference in locoregional recurrence or survival. While these results suggest that simply identifying patients as node positive will provide adequate information for planning systemic therapy, the results remain inapplicable to patients who have undergone neoadjuvant chemotherapy, those with greater nodal burden than represented in the study, and those patients who will not be receiving radiation, due to planned omission, mastectomy, or contraindications to radiotherapy. In these scenarios, characterizing the sentinel node intraoperatively still merits attention. 

Yet a separate question remains as to whether the type or distribution of metastases is important. Macrometastases by definition are those tumor foci greater than 2 mm. The seventh edition of American Joint Committee on Cancer (AJCC) staging for breast cancer defines micrometastases as tumor deposits greater than 0.2 mm but less than 2 mm and are designated pN1(mic) [12]. Isolated tumor cells (ITC) are defined as clusters of cells no greater than 0.2 mm and designated pN0(i+). The clinical significance of micrometastases and isolated tumor cells continues to be investigated. Recent studies have evaluated the prognostic impact of isolated tumor cells or micrometastases in breast cancer and have found that there was a statistically significant decrease in 5-year disease-free survival rate in women with favorable early-stage breast cancer who did not undergo adjuvant treatment compared to the adjuvant therapy group although the impact that this should exert on clinical decision making remains unclear [13, 14]. 

An understanding of tumor cell distribution and its relationship to lymphatic flow is important to highlight the significance of our findings. Unfiltered lymph fluid flows regionally through afferent lymph channels, traverses the outer capsule through the cortex, and flows through the paracortex to the medulla. The filtered fluid then exits in the lymph node through the efferent channel via the hilum. One study utilizing three-dimensional reconstruction to evaluate metastatic tumor cell distribution in sentinel lymph nodes found metastases to be located at the afferent pole in 17 of 19 tumor-involved sentinel nodes [15]. In seven nodes, metastases were confined to the afferent pole, with the balance containing metastases extending to the efferent pole. However, only two cases displayed metastases confined to the efferent pole. These findings correlate with our study results, showing that the afferent pole contained the majority of metastases. In addition, the hotspot was located in the afferent pole on the cortical surface of the node in the majority of both the positive and negative sentinel nodes for our study. We could, therefore, postulate that focusing in the area of the hotspot would improve the sensitivity of nodal analysis.

A limitation of our study is the small sample size. The study spanned just over a year, and the number of involved nodes is low. The standard use of axillary ultrasound paired with fine-needle aspiration biopsy (USFNA) as part of staging new breast cancers at our institution has decreased the number of intraoperatively detected nodal metastases. Based on our previously published data, our institutional policy has been to recommend axillary USFNA for all invasive ductal carcinomas greater that 1.5 cm [16]. This strategy biases the study cohort towards low-volume axillary disease. Nonetheless, there are patients where USFNA is of limited success due to body habitus, patient tolerability, or the receipt of neoadjuvant chemotherapy. 

Notably all patients in this study were hormone receptor positive. This also is likely related to the institutional interest in neoadjuvant chemotherapy trials. Given the documented role for systemic therapy in hormone receptor negative breast cancer, the majority of these patients are treated neoadjuvantly at our institution.

 As the results of ACoSOG Z-0011 modify our current practices, intraoperative nodal analysis may become less prevalent, except in those groups where the trial results are not applicable, such as patients who will not receive whole breast radiotherapy, patients with more than 3 positive sentinel nodes, and patients undergoing mastectomy. As the mastectomy rate has been about 30–40% and is increasing nationally combined with the evolving strategies for less comprehensive breast radiotherapy, our results remain relevant to many patients [17]. Furthermore, understanding the patterns of disease spread may aid in reducing the false negative results in nodal evaluation and allow for more effective utilization of ever tightening resources.

## 5. Conclusion

In patients where the results of intraoperative assessment of the sentinel node will impact proceeding to axillary dissection, focusing the examination to the afferent pole and within 4 mm of the hot spot should enhance intraoperative detection of sentinel node metastasis.

## Figures and Tables

**Table 1:** Patient and tumor characteristics.

	Study population
	N: 12 (100%)
Age (years)	
Mean	55
Range	44–79

Histologic type	
IDC	8 (66.6)
ILC	2 (16.6)
Mixed IDC/ILC	2 (16.6)

Tumor stage	
IIA	6 (50)
IIB	1 (8.3)
IIIA	5(41.6)

Tumor grade	
I	2 (16.6)
II	8 (66.6)
III	1(8.3)*

LVI	
Present	7 (58.3)
Absent	5(41.6)

ER/PR status	
ER and/or PR positive	12 (100)
ER/PR negative	0

Her2neu	1 (8.3)
Positive	11 (91.6)**
Negative	

*One patient had received neoadjuvant chemotherapy so tumor grade is not available.

**IHC was performed first and if intermediate FISH was done.

IDC: infiltrating ductal carcinoma, ILC: infiltrating lobular carcinoma, LVI: lymphovascular invasion, ER: estrogen receptor, and PR: progesterone receptor.

**Table 2:** Results.

Total Sentinel Nodes	Cortex Hotspot	Hilum Hotspot
Positive (19)	15 (79%)	4 (21%)
Negative (95)	77 (81%)	18 (19%)
